# Evaluation of a Distribution Model to Increase Access to Affordable Fruits and Vegetables

**DOI:** 10.5888/pcd21.230206

**Published:** 2024-01-04

**Authors:** Kerri L. Vasold, Karah Mantinan, Rebecca Hofer, Michael Waddle, Amy Slechta

**Affiliations:** 1 Altarum Institute, Ann Arbor, Michigan; 2 Partnership for a Healthier America, Washington, District of Columbia

## Abstract

**Introduction:**

Identifying effective, sustainable strategies to increase fruit and vegetable consumption is critical to addressing chronic disease risk. Models that provide incentives for produce purchases through reduced-cost or no-cost produce shares are promising. The purpose of our study was to examine the impact on fruit and vegetable intake of Good Food for All, a community-based program to distribute no-cost produce boxes to participants with low incomes. We also assessed program satisfaction and future interest in purchasing an affordable produce box.

**Methods:**

The Good Food for All program was implemented in 22 US cities. Surveys were administered at baseline and postintervention. An online research panel was used as a comparison group and weighted to be demographically comparable to the intervention group. Descriptive statistics and adjusted difference-in-difference (ADID) models were used to examine differences in outcomes between groups.

**Results:**

Respondents (intervention n = 632; comparison n = 1,153) were primarily White, non-Hispanic, and female. Intervention participants had a greater increase in total fruit consumption frequency (ADID: 0.43 times/d; 95% CI, 0.21–0.64; *P* < .001), total vegetable consumption frequency (ADID: 0.52 times/d; 95% CI, 0.12–0.91; *P* = .01), and total fruit and vegetable consumption frequency (ADID: 1.03 times/d; 95% CI, 0.49–1.56; *P* < .001) than comparison respondents. Most intervention participants reported boxes contained the right amount of food (71.9%) and high-quality produce (68.4%) and were willing to purchase a future produce box (85.0%).

**Conclusion:**

Findings indicate that a produce box distribution model increased fruit and vegetable consumption, had high satisfaction among participants, and generated interest in purchasing affordable produce boxes. Future studies should explore feasibility of offering low-cost produce boxes at grocery stores and determine appropriate pricing models to enhance access and sustainability.

**Summary**

**What is already known on this topic?**

Distribution of reduced-cost or no-cost produce boxes is a promising initiative to increase access to and consumption of fruits and vegetables among people with low incomes. Previous work has focused primarily on school-based or community-supported agriculture models.

**What is added by this report?**

Our study used a robust design, including a large sample across 22 cities and a comparison group, to evaluate the effectiveness of a community-based produce box distribution model.

**What are the implications for public health practice?**

Community-based models of produce box distribution are effective at increasing fruit and vegetable consumption, can have high satisfaction rates among participants, and offer a potential marketplace solution to increasing access to affordable produce.

## Introduction

Poor dietary intake is responsible for more deaths globally than any other risk factor (1). Diets high in fruits and vegetables are associated with lower risk of diabetes, cardiovascular disease, obesity, cancer, and all-cause mortality (2–4). Consumption of fruits and vegetables is lower among people with the lowest household incomes (5), who may face barriers to fruit and vegetable consumption, such as cost and taste preferences (6), limited availability, and limited access to these products (7).

Identifying effective, sustainable strategies to improve dietary quality through increasing fruit and vegetable consumption is critical to addressing chronic disease risk. Food-as- medicine models, including produce prescription programs that provide food subsidies in the form of vouchers redeemable at farmers markets or grocers or produce boxes distributed or delivered to participants, have gained popularity in recent years (8,9). These programs typically aim to improve cardiometabolic risk factors and are delivered in coordination with a clinician or health center. Other promising models provide incentives for fruit and vegetable purchases among low-income households through reduced-cost or no-cost produce shares from community-supported agriculture and food cooperatives (10–14). However, barriers in implementation exist. Furthermore, many of these programs are supported by short-term grant funding, which limit their reach and sustainability (15). A marketplace solution that could adapt this approach to the community setting and offer weekly produce distribution at locations where consumers are already shopping for food may ameliorate some of these barriers.

Good Food for All, developed by Partnership for a Healthier America (16), is a 12-week community-based intervention that uses a produce box distribution model to increase access to fresh fruits and vegetables. Each week, participants can receive approximately 50 servings of fresh produce. Partnership for a Healthier America partnered with Altarum to conduct an evaluation of Good Food for All. The purpose of our study was to determine the effectiveness of Good Food for All in increasing fruit and vegetable intake among participants, satisfaction with the program, and willingness to pay for a produce box or other produce-centric meal kits in the future. We hypothesized that Good Food for All participants would significantly increase fruit and vegetable consumption compared with a comparison sample, report high satisfaction with the program, and be interested in purchasing produce boxes or meal kits in the future.

## Methods

### Intervention

Good Food for All was a 12-week community-based initiative that provided boxes of fresh produce to individuals and families with limited financial resources. The program consisted of weekly distribution of fresh fruits and vegetables procured from produce distribution companies and packaged in a Good Food for All box. Each box was designed for a family of 4 with approximately 50 servings of fresh produce, with a focus on a wide variety of high-quality fruits and vegetables. To promote dietary diversity and maximize participant satisfaction, produce box guidelines limited starchy vegetables (eg, corn, white potatoes, lima beans, green peas) to 17.5% of box contents by weight and emphasized dark green vegetables, red and orange vegetables, and fruits. As part of the program, a recipe booklet was also provided that included healthy recipes featuring box items. The program’s primary objective was to increase participants’ fruit and vegetable consumption. Other goals included instilling healthy habits among participants and evaluating the program’s potential as a long-term, sustainable solution to improve food access.

Good Food for All was implemented in 22 cities across 10 midwestern states (Iowa, Illinois, Indiana, Kansas, Michigan, Minnesota, Nebraska, Ohio, Wisconsin, South Dakota) by using a phased rollout design during the Delta and Omicron waves of the COVID-19 pandemic. Most program cities were in metropolitan areas with a population of 1 million or more (50.0%, n = 11) or areas with populations of 250,000 to 1 million (40.9%, n = 9), identified by using the 2013 USDA Economic Research Service Rural–Urban Continuum Codes (https://www.ers.usda.gov/data-products/rural-urban-continuum-codes/). A smaller percentage of program cities were in metropolitan areas of fewer than 250,000 population (4.7%; n = 1) or in nonmetropolitan areas (4.7%, n = 1). The program was launched over the course of approximately 15 weeks, from mid-July 2021 to late October 2021. Produce boxes were distributed for 12 weeks from each city’s program start date.

Partnerships existed or were established with a variety of community-based organizations to facilitate the Good Food for All program in communities with limited resources. Community partners were chosen because they served individuals and families with limited resources. Types of community partners included emergency food distribution sites such as food banks and pantries, city government departments, YMCAs, and other local organizations. This community-based model was designed to reach households with limited financial resources, reduce barriers to access, and promote equity. Partner agencies assisted in recruitment of participants and program deployment, and each city received a funding award in the amount of $25,000 to offset costs associated with their participation. Most community partners also worked with sub-recipients (eg, food banks/pantries, schools) to recruit participants and distribute produce boxes in community-based locations.

To limit barriers to participation, participants did not need to meet any specific eligibility criteria, and registration was not required to receive a box. The program identified households with limited financial resources by recruiting participants through community-based organizations that served this population. Community partners were provided with customizable recruitment materials, including flyers and social media materials, to aid in recruitment.

### Study design

We used a mixed-methods longitudinal study design and a comparison group to assess program impact. A power analysis was conducted that informed project sampling. The study protocol was approved by the Social Solutions International Institutional Review Board.

### Survey protocol

Program participants and comparison group respondents were invited to complete an online survey at baseline and postintervention. Demographic characteristics of survey respondents were collected as part of the baseline survey.

### Intervention group

Intervention group respondents were recruited through community partners in each distribution city throughout the first 4 weeks of the program. Baseline surveys were administered via text message or email to participants on a weekly basis as they enrolled during the first 4 weeks of program implementation. Reminders were sent 1 week after the initial survey invitation. Respondents who completed the survey at baseline were asked to opt in to the postsurvey and provide their preferred contact method (text message or email). The week following the last box distribution in their city (8–12 weeks after baseline survey invitations, based on enrollment date), respondents who opted in were invited to complete the postsurvey. A reminder was sent 1 week after the initial invitation to participate in the postsurvey.

The online surveys were designed to take 10 minutes to complete and were available in English and Spanish. Survey respondents were emailed an electronic $10 gift card 2 to 4 weeks after completion of each survey as a thank-you for their participation.

### Comparison

Comparison group respondents were recruited from an online research panel. The online panel consisted of a nationally representative, US census–balanced sample that allowed for the selection of respondents who reflected the target population. The parameters for participation in the survey were an annual household income under $50,000 and permission to recontact for the postintervention survey. A national sample was used for the comparison group because a Midwest-only sample did not provide a sufficient sample size. Online panel participants typically remain on the panel for less than 2 months; to maximize response rate and ensure sufficient sample size, a 6-week follow-up period was used in the comparison group.

### Data collection measures

Primary outcome measures were related to changes in fruit and vegetable consumption from baseline to postintervention. At each time point, fruit and vegetable consumption was measured by using a subset of questions from the National Cancer Institute (NCI) All-Day Screener (17). The NCI screener was shown to be useful for estimating median dietary intakes of US populations (18). The original screener consists of 19 questions to assess frequency and quantity of fruit and vegetable intake (17). A subset of 10 measures that assess intake frequency were adopted for our study to minimize respondent burden and align with equitable evaluation practices. Reporting serving size can be challenging for respondents, and measuring consumption frequency is a method used in evaluation of audiences with low incomes to estimate overall consumption (19). Each of the 10 measures had 10 frequency category choices that ranged from never to 5 or more times per day in response to the following question: “Over the last month, how many times per month, week, or day did you eat fruit? Count any kind of fruit — fresh, canned, and frozen. Do not count juices. Include fruit you ate at all mealtimes and for snacks.” NCI screener questions about serving size such as “Each time you ate fruit, how much did you usually eat?” were not included. Intake frequency was calculated as described by NCI’s All-Day Screener scoring guidelines.

### Statistical analysis

Data cleaning included removal of survey respondents outside of the recommended time period (ie, 2 weeks after receiving the survey) based on the phased rollout design. Additionally, respondents who did not complete most of the survey were removed from analyses (baseline, n = 368; post, n = 23).

Comparison sample data were weighted to the intervention sample characteristics by using race, age, sex, and federal poverty level status. We used SAS-callable SUDAAN’s PROC WTADJUST procedure (RTI International) to apply poststratification weights to the sample with the intervention sample weighted to 1.0. The mean weight value was 5.8 with a median weight of 0.5. Weighted data were used to conduct analyses between the intervention and comparison samples.

Descriptive statistics such as frequencies and means were calculated for all variables of interest. Mean comparisons and adjusted difference-in-difference (ADID) models were used to examine differences in outcomes over time and between groups. When relevant, models were adjusted for program participation levels, race, federal poverty level, age, and whether children were in the household. All analyses were conducted in IBM SPSS Statistics version 27 (IBM Corp) and SAS version 9.4 (SAS Institute), and significance was determined at α = .05.

## Results

### Demographic characteristics

Our total analytic sample consisted of 632 intervention group respondents and 1,153 comparison group respondents. Among intervention group respondents, the baseline survey had a 14% response rate, and the postsurvey had a 44% response rate based on those respondents who opted in to receiving the postsurvey. Most intervention respondents (n = 551; 87.2%) completed the postsurvey within 1 week of receiving their last produce box. The comparison group had a 40% response rate to the postsurvey. A baseline response rate was not available for the comparison group because data were collected via web panel. Intervention respondents were primarily White (61.9%), non-Hispanic, non-Latino or non-Latina (88.0%), and female (91.4%) (Table 1). The average household size was 3.9 people; 55.9% were from households with children, 47.0% had a household income below 130% of the FPL, and 66.0% participated in 1 or more assistance programs. Approximately one-half (50.8%) of intervention respondents picked up their produce boxes for 10 to 12 weeks of the program. Comparison group respondents were similar, with respondents primarily White (54.1%); non-Hispanic or non-Latino or non-Latina (92.0%), and female (90.4%). Additionally, 48.9% of comparison respondents were from households with children, 51.4% had a household income below 130% of the FPL, and 40% participated in 1 or more assistance programs, including both food and nonfood–based programs. During this time, many assistance programs were expanded because of pandemic relief responses.

**Table 1:** Demographic Characteristics, Participants in Good Food for All Produce Box Distribution Model in 22 US Cities, 2021^a^^,^ b

Demographic characteristic	Intervention group (n = 632), no. (%)	Comparison group (n = 1,153), no. (%)
**Race**		
American Indian or Alaskan Native	5 (0.8)	3 (0.3)
Asian	18 (2.9)	99 (8.7)
Black or African American	155 (25.0)	383 (33.6)
Native Hawaiian or Other Pacific Islander	1 (0.2)	0
White	383 (61.9)	617 (54.1)
Two or more races	22 (3.6)	39 (3.4)
Other race	14 (2.3)	0
Don’t know	21 (3.4)	0
**Hispanic, Latino or Latina background**		
Hispanic, Latino or Latina	68 (10.8)	88 (7.5)
Non-Hispanic, non-Latino or non-Latina	555 (88.0)	1,073 (92.0)
Don’t know	8 (1.3)	5 (0.4)
**Sex**		
Female	573 (91.4)	1,050 (90.4)
Male	46 (7.3)	108 (9.3)
Other	1 (0.2)	3 (0.3)
Prefer not to answer	7 (1.1)	1 (0.1)
**Age, y**		
18–24	15 (2.4)	40 (3.4)
25–34	135 (21.4)	112 (9.6)
35–44	212 (33.6)	468 (40.1)
45–54	112 (17.7)	237 (20.3)
55–64	89 (14.1)	63 (5.4)
65–74	58 (9.2)	177 (15.2)
75 or older	5 (0.8)	66 (5.7)
Prefer not to answer	5 (0.8)	4 (0.3)
**Household composition**		
Respondent only	44 (7.0)	223 (19.0)
Household with children	353 (55.9)	574 (48.9)
Household with multiple adults and no children	235 (37.2)	376 (32.1)
**Federal poverty level status**		
Above approximately 130% of FPL	335 (53.0)	570 (48.6)
Below approximately 130% of FPL	297 (47.0)	603 (51.4)
**Participation in assistance programs^c^**		
SNAP/EBT, Child Tax Credit, Earned Income Tax Credit, Supplemental Security Income, or other cash assistance or tax credit programs	192 (46.0)	249 (52.6)
WIC (Free or reduced-price school lunch or breakfast)	102 (24.5)	68 (14.4)
Free or reduced-price school lunch or breakfast	191 (45.8)	128 (27.0)
Free summer meals	107 (25.7)	32 (6.8)
Head Start	27 (6.5)	48 (10.2)
Food pantry	155 (37.2)	50 (10.5)
Medicaid	206 (49.4)	263 (55.5)
TANF, Child Tax Credit, Earned Income Tax Credit, Supplemental Security Income, or other cash assistance or tax credit programs	46 (11.0)	49 (10.4)
I/we do not participate in any of these programs	215 (34.0)	699 (59.6)
**Number of boxes picked up at intervals, weeks**		
0	28 (4.7)	—
1–3	88 (14.8)	—
4–6	93 (15.7)	—
7–9	83 (14.0)	—
10–12	302 (50.8)	—

Abbreviations: —, not applicable; FPL, federal poverty level; SNAP/EBT, Supplemental Nutrition Assistance Program/Electronic Benefits Transfer; TANF, Temporary Assistance for Needy Families; WIC, Special Supplemental Nutrition Program for Women, Infants, and Children.

a Cities were located in the following 10 midwestern states: Iowa, Illinois, Indiana, Kansas, Michigan, Minnesota, Nebraska, Ohio, Wisconsin, South Dakota. Most program cities were located in metropolitan areas with a population of 1 million or more (50.0%, n = 11) or areas with populations of 250,000 to 1 million (40.9%, n = 9), identified by using the 2013 USDA Economic Research Service Rural–Urban Continuum Codes. A smaller percentage of program cities were in metropolitan areas of fewer than 250,000 population (4.5%; n = 1) or in nonmetropolitan areas (4.5%, n = 1).

b The program was launched over the course of approximately 15 weeks, from mid-July 2021 to late October 2021. Produce boxes were distributed for 12 weeks from each city’s program start date.

c Respondents were asked to select all assistance programs that they participated in, so columns may not total to 100%.

### Fruit and vegetable consumption frequency

After adjusting for covariates, from baseline to postintervention, intervention respondents had a greater increase in total fruit consumption frequency (ADID: 0.43 times/d; 95% CI, 0.21–0.64, *P* < .001), total vegetable consumption frequency (ADID: 0.52 times/d, 95% CI, 0.12–0.91, *P* = .01), and total fruit and vegetable consumption frequency (ADID: 1.03 times/d; 95% CI, 0.49–1.56, *P* < .001) than comparison respondents (Table 2).

**Table 2:** Difference in Differences in Frequency of Consumption (Times per Day) Between Participants in Intervention and Comparison Groups, from Baseline to Postintervention^a^^,^ b, Good Food for All Produce Box Distribution Model in 22 US Cities, 2021^c^

Variable	Intervention group (n = 632)			Comparison group (n = 1,153)			ADID	
	Baseline mean (SD)	Post mean (SD)	Difference mean (SD)	Baseline mean (SD)	Post mean (SD)	Difference, mean (SD)	DID (95% CI)^a ^	*P* value^d^
Combined fruit and vegetables	3.93 (3.48)	4.57 (4.04)	0.65 (0.15)	3.72 (4.34)	3.40 (3.35)	−0.34 (0.34)	1.03 (0.49–1.56)	<.001
Fruit	1.64 (1.66)	1.88 (1.89)	0.24 (0.08)	1.48 (1.56)	1.32 (1.27)	−0.18 (0.11)	0.43 (0.21–0.64)	<.001
Vegetables	2.31 (2.28)	2.69 (2.65)	0.39 (0.10)	2.24 (3.32)	2.15 (2.71)	−0.22 (0.26)	0.52 (0.12–0.91)	.01

Abbreviations: ADID: adjusted difference in differences; DID, difference in differences.

a Adjusted for race (Black or African American, White, all other races), age (18–54 y, ≥55 y), children in the household (yes, no), federal poverty status (above or at/below 130% FPL).

b Cities were located in the following 10 midwestern states: Iowa, Illinois, Indiana, Kansas, Michigan, Minnesota, Nebraska, Ohio, Wisconsin, South Dakota. Most program cities were located in metropolitan areas with a population of 1 million or more (50.0%, n = 11) or areas with populations of 250,000 to 1 million (40.9%, n = 9), by using the 2013 USDA Economic Research Service Rural–Urban Continuum Codes. A smaller percentage of program cities were in metropolitan areas of fewer than 250,000 population (4.5%; n = 1) or in nonmetropolitan areas (4.5%, n = 1).

c The program was launched over the course of approximately 15 weeks, from mid-July 2021 to late October 2021. Produce boxes were distributed for 12 weeks from each city’s program start date.

d Significance determined by ADID where α = .05.

Among intervention group respondents, differences in fruit and vegetable consumption frequency (times/d) were observed based on self-reported participation level in the program. Intervention group respondents who reported picking up produce boxes for 1 to 6 weeks did not experience significant change in fruit or vegetable consumption frequency from baseline to postintervention (Table 3). Respondents who reported picking up fruit boxes for 7 to 9 weeks reported a significant increase in fruit consumption frequency from baseline (times/d: mean, 1.52; SD, 1.39) to postintervention (times/d: mean, 1.99; SD, 1.93) (*P* = .03). Respondents who reported picking up produce boxes for 10 to 12 weeks reported a significant increase in fruit consumption frequency from baseline (times/d: mean, 1.57; SD, 1.50) to postintervention (times/d: mean, 1.79; SD, 1.67) (*P* = .003) and vegetable consumption frequency from baseline (times/d: mean, 2.23; SD, 1.66) to postintervention (times/d: mean, 2.87; SD, 2.86) (*P* < .001).

**Table 3:** Frequency of Fruit and Vegetable Consumption by Participation Level Among Intervention Group Participants (N = 632), Good Food for All Produce Box Distribution Model in 22 US Cities, 2021^a^^,^ b

Variable	No. of respondents	Baseline, mean (SD)	Post, mean (SD)	*P* value
**Fruit**				
1 to 6 weeks	179	1.78 (1.84)	1.77 (1.82)	.80
7 to 9 weeks	82	1.52 (1.39)	1.99 (1.93)	.03^c^
10 to 12 weeks	300	1.57 (1.50)	1.79 (1.67)	.003^c^
**Vegetables**				
1 to 6 weeks	172	2.39 (2.99)	2.52 (2.24)	.20
7 to 9 weeks	81	2.31 (2.23)	2.33 (1.57)	.23
10 to 12 weeks	296	2.23 (1.66)	2.87 (2.86)	<.001^c^

a Cities were located in the following 10 midwestern states: Iowa, Illinois, Indiana, Kansas, Michigan, Minnesota, Nebraska, Ohio, Wisconsin, South Dakota. Most program cities were located in metropolitan areas with a population of 1 million or more (50.0%, n = 11) or areas with populations of 250,000 to 1 million (40.9%, n = 9), identified by using the 2013 USDA Economic Research Service Rural–Urban Continuum Codes. A smaller percentage of program cities were in metropolitan areas of fewer than 250,000 population (4.5%; n = 1) or in nonmetropolitan areas (4.5%, n = 1).

b The program was launched over the course of approximately 15 weeks, from mid-July 2021 to late October 2021. Produce boxes were distributed for 12 weeks from each city’s program start date.

c Significance determined by Related-Samples Wilcoxon Signed Rank Test where α = .05.

### Program satisfaction

Most intervention respondents when asked about their perceptions of the quality and amount of produce they received in their produce boxes said produce quality was high (68.4%) and boxes contained the right amount of food despite different household sizes (71.9%) (Table 4)**.**

**Table 4:** Program Satisfaction Among Intervention Group Participants (n = 632), Good Food for All Produce Box Distribution Model in 22 US Cities, 2021^a^^,^ b

Variable	n (%)
**Quality**	
High	386 (68.4)
Neither high nor low	135 (23.9)
Low	43 (7.6)
**Amount**	
Too much food	93 (16.5)
Right amount of food	406 (71.9)
Too little food	66 (11.7)

a Cities were located in the following 10 midwestern states: Iowa, Illinois, Indiana, Kansas, Michigan, Minnesota, Nebraska, Ohio, Wisconsin, South Dakota. Most program cities were located in metropolitan areas with a population of 1 million or more (50.0%, n = 11) or areas with populations of 250,000 to 1 million (40.9%, n = 9), identified by using the 2013 USDA Economic Research Service Rural–Urban Continuum Codes. A smaller percentage of program cities were in metropolitan areas of fewer than 250,000 population (4.5%; n = 1) or in nonmetropolitan areas (4.5%, n = 1).

b The program was launched over the course of approximately 15 weeks, from mid-July 2021 to late October 2021. Produce boxes were distributed for 12 weeks from each city’s program start date.

### Willingness to purchase

Most respondents in both the intervention and comparison groups were willing to purchase a produce box or meal kit in the future. In the intervention group, 85.0% were willing to purchase a produce box and 62.8% were willing to purchase a meal kit. In the comparison group, 87.9% of respondents were willing to purchase a produce box and 66.3% were willing to purchase a meal kit (Table 5). 

**Table 5:** Willingness to Purchase Produce Boxes and Meal Kits at Post-Intervention Among Participants, Good Food for All Produce Box Distribution Model in 22 US Cities, 2021^a^^,^ b

Variable	Intervention group (n = 632), n (%)	Comparison group (n = 1,153), n (%)
**Produce boxes, $**		
<5	91 (14.4)	182 (15.6)
5 to 9.99	267 (42.4)	382 (32.8)
10–14.99	121 (19.2)	239 (20.5)
15 –19.99	38 (6.0)	126 (10.8)
≥20	19 (3.0)	95 (8.1)
Not willing to purchase	94 (14.9)	142 (12.2)
**Meal kits, $**		
<30	282 (44.7)	500 (43.1)
30 –39.99	78 (12.4)	192 (16.5)
40–49.99	24 (3.8)	65 (5.6)
50–54.99	9 (1.4)	11 (1.0)
≥55	3 (0.5)	1 (0.1)
Not willing to purchase	235 (37.2)	391 (33.7)

a Cities were located in the following 10 midwestern states: Iowa, Illinois, Indiana, Kansas, Michigan, Minnesota, Nebraska, Ohio, Wisconsin, South Dakota. Most program cities were located in metropolitan areas with a population of 1 million or more (50.0%, n = 11) or areas with populations of 250,000 to 1 million (40.9%, n = 9), identified by using the 2013 USDA Economic Research Service Rural–Urban Continuum Codes. A smaller percentage of program cities were in metropolitan areas of fewer than 250,000 population (4.5%; n = 1) or in nonmetropolitan areas (4.5%, n = 1).

b The program was launched over the course of approximately 15 weeks, from mid-July 2021 to late October 2021. Produce boxes were distributed for 12 weeks from each city’s program start date.

## Discussion

We evaluated the effectiveness of the Good Food for All program in increasing fruit and vegetable intake among participants, their satisfaction with the program, and their willingness to pay for a produce box or produce-centric meal kits in the future. Overall findings indicate that participation in Good Food for All led to a significantly greater increase in frequency of fruit, vegetable, and combined fruit and vegetable consumption (times/d) among intervention group respondents relative to the comparison group. Among intervention group respondents, significant increases in fruit and vegetable consumption were related to program participation levels. Results suggest that higher program fidelity is associated with positive changes in fruit and vegetable consumption frequency and that participation in less than half of the program is not sufficient exposure to the intervention to lead to behavior change. Additionally, intervention respondents had a high satisfaction with the produce box contents in terms of amount provided and quality of the produce. Lastly, most intervention respondents and comparison group respondents also indicated an interest in purchasing produce boxes or meal kits in the future, supporting an interest in the overall distribution model.

Although different settings and pricing models were used, overall findings of Good Food for All align with previous literature related to fruit and vegetable consumption and future purchasing interests. Results of our study are similar to previous cost-offset community-supported agriculture and produce prescription studies where participants receiving produce boxes reported either increased fruit and vegetable intake from baseline to postintervention or higher levels of fruit and vegetable intake than comparison groups (9,12–14). In the evaluation of Brighter Bites, a school-based program that offered weekly produce distributions paired with nutrition education, significant increases in fruit and vegetable consumption occurred among children but varied among adult participants (10). However, a longitudinal 2-year follow-up study of the Brighter Bites program indicated increased fruit and vegetable consumption among child and adult participants between baseline and follow-up (11). A previous study by McGuirt and colleagues also noted a similar pricing range for produce boxes, with most participants interested in paying $15 or less for a produce box (20).

The sustainability of produce box distribution programs should also be considered. Models such as Good Food for All require funding to pack and ship produce boxes and resources for storage and distribution and may incur costs resulting from food loss or other unanticipated risks. Brighter Bites, which procured donated produce from a food bank, estimated the cost at $2.65 per family per week (8). To the authors’ knowledge, no research exists estimating the costs of produce box programs that use a community distribution model such as Good Food for All, which used produce distribution companies. More research is needed to determine how this cost point could sustain a marketplace solution without sacrificing participant satisfaction or introducing access barriers.

### Strengths

Strengths of this study include the use of validated tools where possible for surveys, a pre–post matched pair design, and the use of a national comparison sample. Our study also included a large sample size with participants from 22 cities across the Midwest. Additionally, the ability to conduct adjusted analyses to take into consideration confounding variables in relationships supported validity of results. A strength of overall program implementation included partnering with trusted community organizations in each city to distribute produce boxes in locations known to and used by participants.

### Limitations

Limitations of this study include respondent bias resulting from the dissemination method of the survey and participant incentives. Our study had a low baseline response rate despite the inclusion of an incentive. Thus, because not everyone who participated in the program responded to the survey, nonresponse bias may have affected the generalizability of study findings. We did not offer a mailed paper option to complete the survey, which could have limited possible participation for those without access to texting or email. Additionally, generalizability of study findings may be limited because both intervention and comparison group respondents were primarily White, non-Hispanic, non-Latino or non-Latina, and female. It is also unknown whether the demographics of the intervention respondent group align with the demographics of the program participants because this information was not captured in program enrollment. Recall bias is also a limitation because data on program participation and fruit and vegetable consumption were collected via self-report. Nutrition intervention studies that rely on self-report are also susceptible to social desirability bias, which may have influenced reported fruit and vegetable consumption among intervention group participants (21). Program implementation also had some initial challenges with distribution and produce quality, which have been noted by previous studies (22). Issues were quickly resolved but may have affected program participation. Lastly, participant behaviors may have been influenced by the COVID-19 pandemic and various pandemic response efforts that were still in place at the time of this study.

### Conclusions

Overall, Good Food for All was effective in supporting families in increasing frequency of consumption of fresh fruits and vegetables through a produce box distribution model, and participants indicated interest in purchasing produce boxes and meal kits in the future. Findings can be applied to future implementation to scale and tailor the Good Food for All program or similar programs that support food equity. Future research should explore how produce boxes or produce-centric meal kits could be made available at other community locations and could test pricing models that are attractive and feasible for people with insufficient resources for regularly accessing and purchasing fruits and vegetables. Additionally, future studies should measure nutrition insecurity and the effects of programs like Good Food for All on rates of nutrition insecurity among people with low incomes.
